# A bi-objective blood supply chain model under uncertain donation, demand, capacity and cost: a robust possibilistic-necessity approach

**DOI:** 10.1007/s12351-022-00710-4

**Published:** 2022-05-12

**Authors:** Javid Ghahremani-Nahr, Ramez Kian, Ehsan Sabet, Vahid Akbari

**Affiliations:** 1 Academic Center for Education, Culture and Research (ACECR), Tabriz, Iran; 2 Nottingham Business School, Nottingham Trent University, Nottingham, NG1 4FQ UK; 3 Wolfson School of Mechanical, Electrical and Manufacturing Engineering, Loughborough University, Leicestershire, LE11 3TU UK; 4 Nottingham University Business School, University of Nottingham, Jubilee Campus, Nottingham, NG8 1BB UK

**Keywords:** Blood supply-chain, Multi-objective, Robust, Fuzzy, Possibilistic–Necessity model

## Abstract

This paper addresses a multi-objective blood supply chain network design, considering economic and environmental aspects. The objective of this model is to simultaneously minimize a blood supply chain operational cost and its logistical carbon footprint. In order to embed the uncertainty of transportation costs, blood demand, capacity of facilities and carbon emission, a novel robust possibilistic-necessity optimization used regarding a hybrid optimistic-pessimistic form. For solving our bi-objective model, three multi-objective decision making approaches including LP-metric, Goal-Programming and Torabi- Hassini methods are examined. These approaches are assessed and ranked with respect to several attributes using a statistical test and TOPSIS method. Our proposed model can accommodate a wide range of decision-makers’ viewpoints with the normalized objective weights, both at the operational or strategic level. The trade-offs between the cost and carbon emission for each method has been depicted in our analyses and a Pareto frontier is determined, using a real case study data of 21 cities in the North-West of Iran considering a 12-month implementation time window.

## Introduction

The blood supply chain (BSC) manages the flow of blood products from donors to patients. It includes the whole blood as well as its components, comprising red blood cells (RBCs), plasma and, platelets (Hosseini-Motlagh et al. 2020a). From the managerial point of view, the main operations in BSC are collection, transportation, testing, component processing, inventory management, and transfusion (Pirabán et al. 2019). These operations necessitate a consistent flow through different entities of the supply chain such as donation/collection centers, blood centers, testing laboratories, storage facilities, distribution centers and demand points. These entities form the different echelons of the supply chain map where its upstream starts with donation centers and its downstream ends with the demand points. Sometimes blood product transshipment is occurred within the same entities when for example, products are sent from a local blood facility (responsible for testing and producing products) to a regional blood facility (also responsible for testing and producing products) for further examination, as there are better facilities available in regional blood centers (see Hosseini-Motlagh et al. 2020a; Wang and Chen 2020; Zhou et al. 2021; Dehghani et al. 2021).

According to the International Society of blood transfusion, the main factors in managing the BSC are the number of regular donors, seasonal influence in demand and supply, precise annual demand forecast, and the awareness of clinicians for appropriate ordering and transfusion of blood units to provide sufficient stock, yet avoid wastage (ISBT 2020). Hence, optimizing the process and network of BSC are crucial where the main goal is generally to satisfy demand at minimal cost. Due to the need to preserve vitality of the blood product, agility and responsiveness is the other main goal, especially under emergency settings. In contrast to emergency services, when long-term BSC settings are in question, additional contemporary supply chain goals such as sustainability can/should be included in the BSC objectives. In other words, the strategic setting of a BSC cannot afford to ignore the carbon footprint and other environmental measures. Such considerations have emerged in studies since 2018. These objectives, such as swiftness, waste, and carbon emission, have highlighted emerging needs for using multi objective decision making frameworks in BSC network design.

The other challenge in BSC design is supply uncertainty. The blood donation can be fairly irregular, hence the demand has a stochastic nature, and the product has a limited shelf-life, while it also needs very strict laboratory tests and special delivery systems within, or prior to its supply points (Beliën and Forcé 2012). This uncertain environment is classified into three categories: stochastic, unknown and fuzzy. In stochastic models, the probability distribution functions of uncertain parameters are known; whereas in the unknown environment there is no information about the probability distributions of uncertain parameters. The fuzzy environment models the uncertain parameters via fuzzy numbers commonly with two types of fuzzy programming: flexible and possibilistic. The flexible programming carries out right-hand side uncertainties while the possibilistic programming deals with uncertainties both in the objective function coefficients and in constraint coefficients (Pirabán et al. 2019).

In this article, a regional BSC network design in north-western part of Iran is addressed as a case study considering the uncertainty and multiplicity of the goals as well as the approaches by which it is dealt with. The research question is mainly associated with the following strategic or operational decisions which determine the topology of the network.

- The number and locations of the collection points, testing labs and storing centers.
- The capacity and types of the facilities at each center.
- The amount of blood to be stored at each facility at each time period.
- The amount of blood in transit between the centers and facilities at each time period.

The remainder of this article has structured as follows. The next section provides the literature review on recent and similar studies as well as our assumptions, scope and contributions statement. Section 3 provides the mathematical problem statement and the corresponding proposed model together with the robust possibilistic reformulation discussions. Section 4 presents the three common approaches used in this study to deal with multiple objectives functions, whereas Sect. 5 presents the numerical results of applying the proposed model on a real blood supply chain case. Finally, Sect. 6 concludes the paper.

## Literature review

According to the review of Beliën and Forcé (2012), studies on BSC started since 1966 and have been an ongoing research topic since then. In their review, 98 papers are classified based on several criteria such as blood products, solution methodology, hierarchical levels in network topology, studied problems, stochastic and deterministic approaches, exact or heuristic solution procedures and case studies. In another review, Osorio et al. (2015) categorize the published analytical modelling articles for BSC according to the echelons of supply chain. They provide a hierarchical decision process within each of these stages and represent the corresponding published papers and therefore, give a more practical viewpoint. In one of the recent surveys at the time of authoring this paper, Pirabán et al. (2019) present a new taxonomy with a higher level of detail by reviewing a wide range of publications over the last two decades. They show a growing trend of related publications over this time period with a pick in years 2017 and 2018 having 15 and 25 articles, respectively. In another recently published work, Williams et al. (2020) provide a detailed review on the application of quantitative methods for the blood collection process from donors. They analyze the literature with respect to methods, modeling objectives and the planning levels such as strategic, tactical and operational.

Due to the rapid growth of publications in the context of blood supply chain, especially in recent years, it is best to conduct a comparative study and content analysis to establish the state-of-the-art publications. Inspiring from the above-mentioned reviews and some recent articles, this paper classifies the most relevant and recent papers with regards to several attributes: (i) Emergency of the models: whether a post-disaster crisis setting is considered or a long-term situation in regional health services is of assumed; (ii) Objective functions: cost, distance, time, reliability and time are the most common objectives in the existing literature; (iii) Mathematical modeling approach: types of the model such as deterministic, stochastic, fuzzy or other techniques; (iv) Multi-objective approach: $\epsilon$-constraint, Goal Programming, Lexicographical or other approaches are among them; (v) Number of echelons: depending on the context and configuration of the case some of these entities may merge or not exist; (vi) Case study: whether it uses a real-scale scenario parameters or not. These attributes are all summarized in Table 1 and the reviewed papers are presented in a chronological order.

### Blood groups compatibility

In addition to the above-mentioned classification, the nature of blood products adds another attribute as we have different blood groups. This brings an extra complexity to the modeling, which has been addressed only in a few studies. For instance, Ghorashi et al. (2020) studied a location-allocation model under emergency situation and compatibility of blood groups. Their model aimed to optimize the cost, time and reliability of routes and their solution approach employs a meta-heuristic algorithm. Hosseini-Motlagh et al. (2020b) considered also perishability and age-based characteristic of the blood beside the substitutability of different groups. Their model addressed the uncertainty in distribution and inventory management under the disaster situation. Similarly, Asadpour et al. (2021) considered the expiration day and blood groups in a BSC network design aiming cost and environmental factors in objectives. Chen and Wang (2019) and Ma et al. (2019) both considered blood type compatibility again, in the context of disaster relief operations. Under a similar setting, the compatibility in transfusion is considered in Alizadeh et al. (2020) and Cheraghi and Hosseini-Motlagh (2020) where both studies proposed bi-objective models with cost objective in common, while the second objectives are respectively time and shortage.

### Type of collection centers Studies

In the BSC network design are mainly focused on location-allocation problems and all the following cited articles have this aim in common. Among the early operations research works, Daskin et al. (2002) and Shen et al. (2003) have proposed single period nonlinear mixed-integer location-inventory models with a series of meta-heuristic solution approaches for their models. However, collection centers can be temporary and even mobile. In the presence of mobile collection centers, the routing is another inherent aspect of the problem. Habibi-Kouchaksaraei et al. (2018) and Eskandari-Khanghahi et al. (2018) have considered the temporary and mobile collection centers, respectively. For further examples of mobile facility and location-routing models in this context the readers may refer to Karadağ et al. (2021), Razavi et al. (2021) and Chaiwuttisak et al. (2014).

### Long-term basis

 In seminal studies in the long-term basis, Şahin et al. (2007) consider a single period location-allocation problem for a network of blood centers including fixed and mobile facilities aiming to categorize the blood level in each center. In another single objective model, Zahiri et al. (2015) discuss a collection and distribution network of blood with a mixed integer mathematical programming model to optimize the flow of blood products between the fixed and mobile facilities. They consider uncertainty of parameters and provide a robust possibilistic programming approach which is the closest study to ours with respect to mathematical modeling approach. In a recent single objective study, Hosseini-Motlagh et al. (2020) have addressed donors motivation and the reliability and robustness of the network alongside the location, and developed a hybrid possibilistic-flexible robust optimization model wherein flexible programming deals with violation of constraints while possibilistic programming handles imprecise parameters. Nagurney et al. (2012) additionally incorporate the shortage risk in the objective and consider the stochastic nature of the supply by optimizing the deterministic equivalent of the risk. They conduct a sensitivity analysis over the unit shortage penalty cost. Arvan et al. (2015), Zahiri and Pishvaee (2017), Zahiri et al. (2018) and Hosseini-Motlagh et al. (2020a) study bi-objective mathematical models in long-term basis as well while they all have the cost element in common. In particular, Arvan et al. (2015) consider the time factor as the second objective and conduct a sensitivity analysis between objectives using the $\epsilon$-constraint method over their deterministic model. On the other hand, the uncertainty is subsumed in Zahiri and Pishvaee (2017) by considering fuzzy parameters and employing a fuzzy possibilistic model, in Zahiri et al. (2018) by applying a multi-stage stochastic programming, and in Hosseini-Motlagh et al. (2020a) by developing a two-stage stochastic programming. The delivery time, unsatisfied demand and substitution levels are respectively addressed as the second objective in these papers.

Among the studies with more than two objectives, Heidari-Fathian and Pasandideh (2018) consider product waste and environmental impacts in addition to cost minimization, which is close to our work. They develop a robust optimization model and the bounded objective function method, in which the most important objective is kept, while the rest are converted to constraints within their desired lower and upper bounds. This is applied to deal with the multi-objective mathematical model. In another multi-objective study, Samani et al. (2019) consider the qualitative aspect regarding capabilities of collection centers besides the cost and freshness objectives. They tackle the uncertainty issue by developing a robust model and apply the interactive Torabi-Hassini method to deal with multiple objective.

### Under emergency/crisis

 In several studies the BSC is modeled for a post-disaster situations. They have similar attributes to the studies under a regular basis. Namely, some objective functions, modeling and solution methodologies, and network typologies are the common factors between them. Among which, Sha and Huang (2012) propose a deterministic multi-period location-allocation problem of emergency blood supply. Their single-objective model minimizes the total operational cost which is solved via a Lagrangian heuristic algorithm. They employ their model on a real case for earthquake situation in China.

In another single-objective study, Jabbarzadeh et al. (2014) investigate a BSC network design of facilities in a post disaster and uncertain situation and propose a robust model to minimize blood transfer costs to hospitals. Rahmani (2019) and Salehi et al. (2019) respectively apply robust modelling and robust stochastic mathematical programming.

Similarly, Samani and Hosseini-Motlagh (2020) employ a robust model which incorporates donors’behavior and their preference over donation facilities, estimation of injuries under several disaster scenarios, uncertainty of parameters and remaining capacity.

In multi-objective studies, the time factor is more frequent in objective functions due to urgency of the blood products under crisis situation (see Fahimnia et al. 2017; Fazli-Khalaf et al. 2017; Samani et al. 2018; Khalilpourazari and Khamseh 2019; Khalilpourazari et al. 2019) whereas cost is the common objective function in all of them. Moreover, the demand coverage is the next common objective (see Kohneh et al. 2016; Habibi-Kouchaksaraei et al. 2018; Samani et al. 2018) while environmental and social effects also exist in the literature (see Eskandari-Khanghahi et al. 2018). Recently, Haghjoo et al. (2020) propose a dynamic location-allocation model under facility disruption whose severity depends on the initial investment, while Shirazi et al. (2021) study a four-echelon supply network for plasma collection and distribution for COVID-19 patients. They have proposed a simulation and optimization model with two objectives including cost and flow time.

### Solution approaches

 The most commonly applied methodologies among the reviewed papers are deterministic modeling, two-stage or multi-stage stochastic programming, possibilistic and robust fuzzy optimizations. They are mainly solved by off-the-shelf optimization packages. However, some have proposed specific heuristic or generic meta-heuristic algorithms (see for eg. Ghorashi et al. 2020; Goodarzian et al. 2021; Haghjoo et al. 2020; Shirazi et al. 2021). In addition, as the majority (nearly 70%) of studies listed in Table 1 are multi-objective, it is worthwhile briefly discussing some of them as summarized in Khalilpourazari and Khamseh (2019): *LP-metric* obtains a solution which minimizes the deviation of the objective functions from their ideal solutions, whilst *Max-Min* maximizes the minimum amount of the objective functions divided by their ideal solutions. In the *Utility function* method, a normalized weight is assigned to each objective function. Then the sum of weighted objective functions is minimized. In both *Goal Attainment (GA)* and *Goal Programming (GP)* methods, first a goal vector is determined, and then the weighted deviation from the determined goals is minimized with respect to the importance of objectives for GA, while the negative and positive deviations from the determined goals are minimized for GP. Finally, in *Torabi-Hassini (TH)* method the deviations of objectives from their goals are first normalized by a membership function and then their weighted summation is minimized.

Our contributions, similarities and differences from the current literature are highlighted in the following subsection and Table 1.

### Contribution statement

Our contribution is fourfold: (i) A bi-objective supply network design is considered. It consists of two objectives: minimizing the operational and investment costs; and minimizing the environmental effect. The sustainability issue in BSC is quite new in the literature and further studies and real cases are needed to highlight the environmental consideration in health sector. (ii) A fuzzy mathematical modelling is employed to tackle the uncertain nature of some factors in our problem. The application of fuzzy models in the context of BSC is ample in the literature and a handful of which are cited above. However, this article proposes a combination of possibilistic and necessity models reflecting a hybrid optimistic-pessimistic viewpoint as its main novelty. Furthermore, the robust counterpart of the proposed model is also formulated and then it is linearized in order to be handled via conventional optimization packages.

(iii) Using a multiple comparison statistical test, three multi-objective decision making techniques including LP-metric, GP and TH methods are rigorously compared. One significant difference of these methods against $\epsilon$-constraint methods in analyzing multiple objectives is that objective functions can be associated with any combination of weights which facilitates a continuous spectrum of performance frontier. In addition, the aforementioned techniques are ranked by the TOPSIS method with respect to seven different attributes. (iv) Finally our model has been inspired by and tested with a real case study in a regional scale. The expert opinion in inputs estimation in our model has been employed to cover unknown and uncertain parameters.

**Table 1:** Summary of the most relevant and recent studies

Paper	Context	Objective(s)	Mathematical modeling approach	Multi-objective approach	# echelons	# periods	Case study
Şahin et al. (2007)	Long-term	Distance	Deterministic MILP	No	1	Single	Yes
Nagurney et al. (2012)	Long-term	Costs, shortage risk	Deterministic equivalent by expected value	Sensitivity analysis	7	Single	No
Sha and Huang (2012)	Emergency (post-disaster)	Cost	Deterministic MILP	No	2	Multiple	No
Jabbarzadeh et al. (2014)	Emergency (post-disaster)	Cost	Robust optimization	No	3	Multiple	Yes
Arvan et al. (2015)	Long-term	Cost, time	Deterministic MILP	Sensitivity analysis, $\epsilon$-constraint	4	Single	No
Zahiri et al. (2015)	Long-term	Cost	Robust possibilistic programming	No	6	Multiple	Yes
Kohneh et al. (2016)	Emergency (post-disaster)	Cost, coverage	Fuzzy possibilistic	Interactive (TH)	5	Multiple	Yes
Fahimnia et al. (2017)	Emergency (post-disaster)	Cost, time	Stochastic	Sensitivity analysis	4	Multiple	No
Fazli-Khalaf et al. (2017)	Emergency (post-disaster)	Cost, time, reliability	Robust fuzzy possibilistic	Single Pareto	5	Multiple	Yes
Ramezanian and Behboodi (2017)	Long-term	Cost	Robust optimization	No	3	Multiple	Yes
Zahiri and Pishvaee (2017)	Long-term	Cost, coverage	Robust fuzzy possibilistic	Sensitivity analysis	5	Multiple	Yes
Eskandari-Khanghahi et al. (2018)	Emergency (post-disaster)	Cost, environmental & social effects	Robust fuzzy possibilistic	$\epsilon$-constraint	4	Multiple	Yes
Habibi-Kouchaksaraei et al. (2018)	Emergency (post-disaster)	Cost, coverage	Robust optimization	Goal programming	3	Multiple	Yes
Heidari-Fathian and Pasandideh (2018)	Long-term	Cost, waste, environmental	Robust, robust optimization	Bounded Objective Function (BOF)	6	Multiple, no	
Samani et al. (2018)	Emergency (post-disaster)	Cost, coverage, time	Two-stage stochastic model	Crisp, interactive (TH)	4	Multiple	Yes
Zahiri et al. (2018)	Long-term	Cost, freshness	Multi-stage stochastic	Multi-objective meta-heuristic	4	Single	Yes
Hamdan and Diabat (2019)	Long-term	Cost, processing time, waste	Two-stage stochastic	$\epsilon$-constraint	4	Multiple	Yes
Khalilpourazari et al. (2019)	Emergency (post-disaster)	Cost, time, demand	Deterministic MILP	Lexicographical weighted Tchebycheff	6	Multiple	Yes
Khalilpourazari and Khamseh (2019)	Emergency (post-disaster)	Cost, time	Deterministic MILP	GP, lexicographical, weighted Tchebycheff, Max-Min, LP-metric, Utility Function, Goal Attainment	4	Multiple	Yes
Armaghan and Pazani (2019)	Emergency (post-disaster)	Cost, reliability of paths	Mixed-integer nonlinear programming	Max-Min, LP-metric, Achieving the Ideal, Utility Function, TOPSIS, Ideal Planning	4	Multiple	Yes
Rahmani (2019)	Emergency (post-disaster)	Cost	Robust optimization	No	3	Multiple	No
Salehi et al. (2019)	Emergency (post-disaster)	Cost	Stochastic and Robust optimization	No	3	Multiple	Yes
Samani and Hosseini-Motlagh (2019)	Long-term/disruption	Cost	Fuzzy analytic hierarchy process	No	4	Multiple	Yes
Samani et al. (2019)	Long-term	Cost, freshness, qualification	Multi-attribute and multi-objective	Interactive (TH)	3	Multiple	Yes
Chen and Wang (2019)	Emergency (post-disaster)	Cost	Two-stage stochastic programming	No	3	Multiple	No
Ma et al. (2019)	Emergency (post-disaster) Emergency (post-disaster)	Unmet demand	mixed-integer	No	3	Single	No
Alizadeh et al. (2020)	Emergency (post-disaster)	Cost, transfusion time	Mixed-integer	Lexicographic	5	Multiple	Yes
Cheraghi and Hosseini-Motlagh (2020)	Emergency (post-disaster)	Cost, shortage	Robust optimization	Interactive (TH)	4	Multiple	Yes
Ghorashi et al. (2020)	Emergency (post-disaster)	Time, cost, reliability	Mixed-integer	Multi-Objective Grey Wolf Optimizer	4	Multiple	Yes
Hosseini-Motlagh et al. (2020)	Long-term	Cost	Possibilistic- flexible robust programming	No	4	Single	Yes
Hosseini-Motlagh et al. (2020a)	Long-term	Cost, substitution	Two-stage stochastic	Interactive (TH)	4	Multiple	Yes
Hosseini-Motlagh et al. (2020b)	Emergency (post-disaster)	Shortage, waste	Robust fuzzy programming	Compromise programming	4	Multiple	Yes
Asadpour et al. (2021)	Long-term	Cost, environmental impact	Mixed-integer	GP	3	Multiple	Yes
Shirazi et al. (2021)	Emergency (COVID-19)	Cost, flow time	Simulation, optimization (deterministic)	$\epsilon$-constraint, SPEA-II, NSGA-II, MOGWO, MOIWO	4	Multiple	Yes
Arani et al. (2021)	Long-term	Environmental impact, social impact, cost	Mixed integer	Scenario-based, multi-choice goal programming	5	Multiple	No
This study	Long-term	Cost, environmental	Robust fuzzy possibilistic-necessity	Goal Programming, Interactive (TH), LP-metric	5	Multiple	Yes

## Problem statement

In this study a regional 4-level supply chain network comprising donors, donation centers, laboratories and blood demand locations is addressed.

**Fig. 1:**
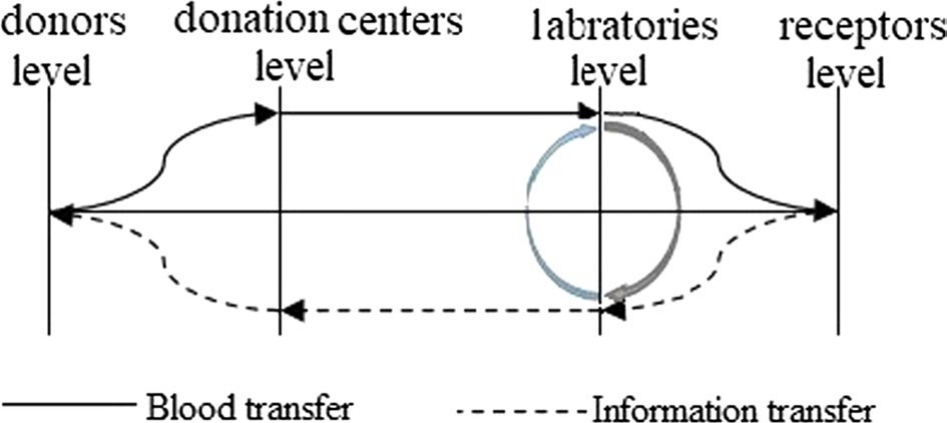
Blood supply chain network configuration.

As illustrated in Fig. 1, once individuals donate at donation centers, the donated blood bags are stored in there adhering to the shelf life to be sent to the laboratories for test. Then, they are distributed from the testing labs to demand points according to their demands. Testing labs can also exchange blood bags between them for testing and distribution purposes if needed. Hence, our addressed problem is in fact a 5-echelon network design. The strategic decision in questions is to determine the number and location of donation centers and testing laboratories aiming to have optimal flow of blood bags and inventories in donation centers and labs, while the tactical goal to minimize (i) Total network costs and (ii) Carbon emission of the logistics. In addition to the fact that the carbon footprint can be reduced by investing more on greener technologies, better insulation and other factors in construction of facilities, the trade-off between the cost and emission in the transportation part of the process originates again from the fact that by investing more on the greener fleet equipment, the per distance unit emission can be reduced. The following assumptions have been made in our blood supply chain network design problem:

- Blood bags can be exchanged between labs for tests and/or distribution purposes.
- The unmet blood demands are penalized.
- To guarantee the coverage of different regions, the distance between the donors ans donation centers are penalized as a coverage cost.
- The transportation cost between facilities, storage costs, time-varying demand amounts, and capacity of the facilities are non-deterministic parameters which are considered as trapezoidal fuzzy parameters.
- The supply chain network is designed over a pre-identified set of locations, denoted by $\mathcal {N}$ where the possible locations for donation centers and laboratories are its subsets, but their exact numbers and locations are to decide.

The nomenclature used for our mathematical model is given in Table 2.

**Table 2:** Sets, parameters and variables notation

Symbol	Definition
Sets	
$\mathcal {I}$	Set of donors locations: $\{1,\ldots ,I\}\subseteq \mathcal {N}$
$\mathcal {J}$	Set of donation centers: $\{1,\ldots ,J\}\subseteq \mathcal {N}$
$\mathcal {K}$	Set of blood laboratories: $\{1,\ldots ,K\}\subseteq \mathcal {N}$
$\mathcal {H}$	Set of blood demand points: $\{1,\ldots ,H\}\subseteq \mathcal {N}$
$\mathcal {G}$	Set of blood groups: $\{1,\ldots ,K\}$
$\mathcal {T}$	Set of time periods: $\{1,\ldots ,T\}$
Parameters	
$v_g$	Shelf time of the blood type $g\in \mathcal {G}$
$\delta _{ij}$	Geographical distance between location $i\in \mathcal {N}$ and $j\in \mathcal {N}$
$f^C_j$	Fixed setup cost of opening blood donation center $j\in \mathcal {J}$
$f^L_k$	Fixed setup cost of opening blood laboratory $k\in \mathcal {K}$
$h^C_j$	Per period unit holding cost of blood packs in donation center $j\in \mathcal {J}$
$h^L_k$	Per period unit holding cost of blood packs in laboratory $k\in \mathcal {K}$
$\theta ^1_{ij}$	Coverage cost from donor $i\in \mathcal {I}$ to donation center $j\in \mathcal {J}$
$\theta ^2_{jk}$	Transportation cost from donation center $j\in \mathcal {J}$ to blood laboratory$k\in \mathcal {K}$
$\theta ^3_{k'k}$	Transportation cost between blood laboratory $k'\in \mathcal {K}$ and $k\in \mathcal {K}$
$\theta ^4_{kh}$	Transportation cost from blood laboratory $k\in \mathcal {K}$ to demand point $h\in \mathcal {H}$
$e^1_{ij}$	Carbon emission in the transit of donor $i\in \mathcal {I}$ to donation center $j\in \mathcal {J}$
$e^2_{jk}$	Carbon emission in the shipment from donation center $j\in \mathcal {J}$ to blood laboratory$k\in \mathcal {K}$
$e^3_{k'k}$	Carbon emission in the shipment between blood laboratory $k'\in \mathcal {K}$ and $k\in \mathcal {K}$
$e^4_{kh}$	Carbon emission in the shipment from blood laboratory $k\in \mathcal {K}$ to demand point $h\in \mathcal {H}$
$d_{hgt}$	Demand for blood of group $g\in \mathcal {G}$ in demand center $h\in \mathcal {H}$ at period $t\in \mathcal {T}$
$\pi _{hgt}$	Penalty cost of unit blood shortage of group $g\in \mathcal {G}$ in demand center $h\in \mathcal {H}$ at period $t\in \mathcal {T}$
$c^C_{jg}$	Maximum capacity for accepting donated blood of group $g\in \mathcal {G}$ in donation center $j\in \mathcal {J}$
$c^L_{kg}$	Maximum capacity for accepting donated blood of group $g\in \mathcal {G}$ in laboratory $k\in \mathcal {K}$
Variable	
$X_{ijgr}$	The amount of type $g\in \mathcal {G}$ blood donated from donor cluster $i\in \mathcal {I}$ in donation center $j\in \mathcal {J}$ at period $r\in \mathcal {T}$
$U_{jkgtr}$	The amount of type $g\in \mathcal {G}$ blood shipped from donation center $j\in \mathcal {J}$ to laboratory $k\in \mathcal {K}$ at period $t\in \mathcal {T}$ which donated in period $r\in \mathcal {T}$ ($r\le t<r+v_g$)
$V_{k'kgtr}$	The amount of type $g\in \mathcal {G}$ blood shipped from laboratory $k'\in \mathcal {K}$ to *K* at period $t\in \mathcal {T}$ which donated in period $r\in \mathcal {T}$ ($r\le t<r+v_g$)
$S_{khgtr}$	The amount of type $g\in \mathcal {G}$ blood shipped from laboratory $k\in \mathcal {K}$ to demand point $h\in \mathcal {H}$ at period $t\in \mathcal {T}$ which donated in period $r\in \mathcal {T}$ ($r\le t<r+v_g$)
$I^C_{jgtr}$	Inventory level of blood type $g\in \mathcal {G}$ donation center $j\in \mathcal {J}$ at period $t\in \mathcal {T}$ which donated in period $r\in \mathcal {T}$ ($r\le t<r+v_g$)
$I^L_{kgtr}$	Inventory level of blood type $g\in \mathcal {G}$ in laboratory $k\in \mathcal {K}$ at period $t\in \mathcal {T}$ which donated in period $r\in \mathcal {T}$ ($r\le t<r+v_g$)
$B_{hgt}$	Unmet demand of blood group $g\in \mathcal {G}$ in demand center $h\in \mathcal {H}$ at period $t\in \mathcal {T}$
$Y^C_{j}$	Binary variable which equals 1 if center *j* is opened; 0, otherwise
$Y^L_{k}$	Binary variable which equals 1 if laboratory *k* is opened; 0, otherwise

### Mathematical programming model of blood supply chain network design.

In the following a mixed integer programming model is provided for the addressed supply network design problem. In the next subsection possibilistic and necessity methods are introduced, and the fuzzy counterpart of this model is developed in order to capture the uncertainty nature of the parameters in reality. Let $r_0:=\max \{1,t+1-v_g\}$.

1 $$\begin{aligned} \text {(MBSC) }\min \omega _1=&\sum \limits _{j\in \mathcal {J}}^{}f^C_j Y^C_j+\sum \limits _{k\in \mathcal {K}}^{}f^L_k Y^L_k +\sum \limits _{t\in \mathcal {T}}\sum \limits _{g\in \mathcal {G}}\Bigg [ \quad \sum _{i,j}\theta ^1_{ij}X_{ijgt} +\sum \limits _{r=r_0}^t\Big ( \sum _{j,k}\theta ^2_{jk}U_{jkgtr}\nonumber \\&\quad +\sum _{k',k}\theta ^3_{k'k}V_{k'kgtr}+\sum _{k,h}\theta ^4_{k'k}S_{khgtr}\Big ) \quad +\sum \limits _{r=r_0}^t\Big (\sum \limits _{j\in \mathcal {J}}h^C_j I^C_{jgtr}+\sum \limits _{k\in \mathcal {K}}h^L_k I^L_{kgtr}\Big )\nonumber \\&\quad +\sum \limits _{h\in \mathcal {H}}\pi _{hgt}B_{hgt} \Bigg ] \end{aligned}$$

2 $$\begin{aligned} \min \omega _2=&\sum \limits _{t\in \mathcal {T}}\sum \limits _{g\in \mathcal {G}}\Big [ \sum _{i,j}e^1_{ij}X_{ijgt} +\sum \limits _{r=r_0}^t\Big (\sum _{j,k}e^2_{jk}U_{jkgtr} +\sum _{k',k}e^3_{k'k}V_{k'kgtr}+\sum _{k,h}e^4_{kh}S_{khgtr}\Big )\Big ] \end{aligned}$$

3 $$\begin{aligned}&\text {s.t.}\nonumber \\&\sum _{r=r_0}^tI^C_{jgtr}=\sum _{r=r_0}^{t-1}I^C_{jg,t-1,r}+\sum \limits _{i\in \mathcal {I}}X_{ijgt}-\sum \limits _{k\in \mathcal {K}}^{}\sum \limits _{r=r_0}^{t}U_{jkgtr}, \quad \forall j,g,t, \nonumber \\&\qquad \qquad \qquad \qquad \text { where }I^C_{..,0,.}:=0, \end{aligned}$$

4 $$\begin{aligned}&I^L_{kgtr}=I^L_{kg,t-1,r}+\sum _{k\in \mathcal {K}}\left( V_{k'kgtr}-V_{kk'gtr}\right) +\sum \limits _{j\in \mathcal {J}}U_{jkgtr}-\sum \limits _{h\in \mathcal {H}}S_{khgtr},\quad \forall k,g,r,t,\nonumber \\&\qquad \qquad \qquad \qquad \text { where } I^L_{..,0,.}:=0, \end{aligned}$$

5 $$\begin{aligned}&\sum \limits _{i\in \mathcal {I}} X_{ijgt}\le c^C_{jg}Y^C_j, \qquad \forall j,g,t, \end{aligned}$$

6 $$\begin{aligned}&\sum \limits _{j\in \mathcal {J}}\sum \limits _{r=r_0}^t U_{jkgtr}+ \sum \limits _{k'\in \mathcal {K}}\sum \limits _{r=r_0}^t V_{k'kgtr}\le c^L_{kg}Y^L_k, \qquad \qquad \qquad \forall k,g,t, \end{aligned}$$

7 $$\begin{aligned}&\sum \limits _{k\in \mathcal {K}}\sum \limits _{r=r_0}^t S_{khgtr}+B_{hgt}\ge d_{hgt}, \qquad \qquad \qquad \forall h,g,t, \end{aligned}$$

8 $$\begin{aligned}&X_{ijgt},U_{jkgtr},S_{khgtr},V_{k'kgtr}\ge 0,\qquad \qquad \qquad \forall i,j,k,g,t,r, \end{aligned}$$

9 $$\begin{aligned}&I^C_{jgtr}, I^L_{kgtr},B_{hgt}\ge 0,\qquad \qquad \qquad \forall j,k,g,t,r, \end{aligned}$$

10 $$\begin{aligned}&Y^C_j,Y^L_k\in \{0,1\},\qquad \forall j, k. \end{aligned}$$

Equation (1) defines the first objective function which corresponds to the total supply chain network design, comprising facility setups, distribution within or between levels, storage in donation centers and labs and finally, unsatisfied demand penalty costs. Equation (2) calculates the total amount of carbon emission caused by distribution operations in the network. Constraints (3) and (4) correspond to blood inventory levels in donation centers and testing labs over the time periods, respectively. Constraints (5) and (6) impose the capacity consideration by limiting the daily amount of blood donation and received blood bags for each blood type in the donation centers and labs, respectively. They also force the model to open a donation center or lab if needed. Constraint (7) corresponds to demand satisfaction where unmet demand is allowed. Finally, (8–10) defines the type and domain of the variables.

In the following subsection the possibilistic-necessity mathematical programming and its robust version are briefly described before reformulating MBSC given above.

### The robust possibilistic-necessity mathematical programming model

In our model the uncertainty is handled by a fuzzy modeling where the uncertain parameters are estimated by decision-makers and field experts within a certain possibility and range. To begin with, let us start with some preliminary building blocks of our model. The trapezoid fuzzy numbers are considered for demand, distribution costs and capacities as shown in Table 3. A trapezoidal fuzzy number $\widetilde{u}=(u_1,u_2,u_3,u_4)$ is associated with a membership function $u(x): \mathbb {R}\rightarrow [0,1]$ as:

$$\begin{aligned} u(x)=\left\{ \begin{array}{ll} \frac{x-u_1}{u_2-u_1},&{} u_1\le x\le u_2,\\ 1, &{} u_2\le x \le u_3,\\ \frac{u_4-x}{u_4-u_3}, &{} u_3,\le x \le u_4,\\ 0, &{} \text {otherwise.} \end{array} \right. \end{aligned}$$

which represents the degree of fuzzy truth. The mechanism by which these numbers are combined, namely defuzzified, with the rest of model is depicted in (20–23) and (28–31).

**Table 3:** Fuzzy parameters of the problem

Trapezoid fuzzy numbers		
$\widetilde{d}_{hgt}=(d^1_{hgt},d^2_{hgt},d^3_{hgt},d^4_{hgt})$		$\widetilde{\theta }^1_{ij}=(\theta ^{1,1}_{ij},\theta ^{1,2}_{ij},\theta ^{1,3}_{ij},\theta ^{1,4}_{ij})$
$\widetilde{c}^C_{jg}=(c^{C,1}_{jg},c^{C,2}_{jg},c^{C,3}_{jg},c^{C,4}_{jg})$		$\widetilde{\theta }^2_{jk}=(\theta ^{2,1}_{jk},\theta ^{2,2}_{jk},\theta ^{2,3}_{jk},\theta ^{2,4}_{jk})$
$\widetilde{c}^L_{kg}=(c^{L,1}_{kg},c^{L,2}_{kg},c^{L,3}_{kg},c^{L,4}_{kg})$		$\widetilde{\theta }^3_{k'k}=(\theta ^{3,1}_{k'k},\theta ^{3,2}_{k'k},\theta ^{3,3}_{k'k},\theta ^{3,4}_{k'k})$
		$$\widetilde{\theta }^4_{kh}=(\theta ^{4,1}_{kh},\theta ^{4,2}_{kh},\theta ^{4,3}_{kh},\theta ^{4,4}_{kh})$$

An efficient approach to manage non-deterministic (possible) constraints which have non-deterministic parameters is the possibilistic chance constraint programming (PCCP) (Pishvaee et al. 2012). In this method a minimum safety margin can be obtained for measuring the confidence level for the constraints satisfaction of those kinds. To this end, commonly two standard fuzzy measures called possibility (POS) and necessity (NEC) are used. The “possibility” offers the optimistic occurrence chance of an event, whereas the “necessity” gives a its pessimistic chance. Therefore, to correctly reflect the logic of a decision-maker in the model (rather than these two extreme measures), a combined possibility-necessity measure is needed. That is, to fairly assume the decision-makers often consider both optimistic and pessimistic chances on these uncertain constraints but decide accordingly (Zahiri et al. 2014). Considering the above-mentioned non-deterministic parameters and employing a possibility-necessity approach, the deterministic equivalent of the BSC model can be formulated. To that end, first consider the following abstract model.

11 $$\begin{aligned}&\min \quad \omega _1=F\mathbf {Y}+C\mathbf {X} \end{aligned}$$

12 $$\begin{aligned}&\min \quad \omega _2=G\mathbf {X} \end{aligned}$$

13 $$\begin{aligned}&\text {s.t.}\nonumber \\&A\mathbf {X}\ge D, \end{aligned}$$

14 $$\begin{aligned}&B\mathbf {X}\le S\mathbf {Y}, \end{aligned}$$

15 $$\begin{aligned}&\mathbf {Y}\in \{0,1\},\quad \mathbf {X}\ge 0, \end{aligned}$$

where *F*, *C*, *G*, *D* and *S* denote the vectors of fixed costs, variable costs, emission amounts, demands and capacities. Additionally, *A* and *B* are the matrices of coefficients, and $\mathbf {X},\mathbf {Y}$ are the continuous and binary variables. It is assumed that *C*, *D* and *S* are vectors of uncertain parameters. Then the basic pessimistic PCCP fuzzy model will be,

16 $$\begin{aligned}&\text {(PM1)\quad }\min \quad E[\omega _1]=F\mathbf {Y}+E[\widetilde{C}]\mathbf {X} \end{aligned}$$

17 $$\begin{aligned}&\min \quad \omega _2=G\mathbf {X} \end{aligned}$$

18 $$\begin{aligned}&\text {s.t.}\nonumber \\&NEC\{A\mathbf {X}\ge \widetilde{D}\}\ge \alpha , \end{aligned}$$

19 $$\begin{aligned}&NEC\{B\mathbf {X}\le \widetilde{S}\mathbf {Y}\}\ge \beta , \end{aligned}$$

$$\begin{aligned} (15), \end{aligned}$$

where $\alpha$ and $\beta$ control the confidence level of non-deterministic constraints satisfaction. Defuzzifying PM1 according to the trapezoidal probability distribution of the uncertain parameters, it can be re-written as below.

20 $$\begin{aligned} \text {(PM2)\quad }\min \quad&\omega _1=F\mathbf {Y}+\left( \frac{C^1+C^2+C^3+C^4}{4}\right) \mathbf {X} \end{aligned}$$

21 $$\begin{aligned} \min \quad&\omega _2=G\mathbf {X} \end{aligned}$$

22 $$\begin{aligned} \text {s.t.}&\nonumber \\&A\mathbf {X}\ge (1-\alpha )D^3+\alpha D^4, \end{aligned}$$

23 $$\begin{aligned}&B\mathbf {X}\le \left( (1-\beta )S^2+\beta S^1\right) \mathbf {Y}, \end{aligned}$$

$$\begin{aligned} (15). \end{aligned}$$

Similarly, for the optimistic case the possibilistic mathematical programming model is as follows,

24 $$\begin{aligned} \text {(OM1)\quad }\min \quad&E[\omega _1]=F\mathbf {Y}+E[\widetilde{C}]\mathbf {X} \end{aligned}$$

25 $$\begin{aligned} \min \quad&\omega _2=G\mathbf {X} \end{aligned}$$

26 $$\begin{aligned} \text {s.t.}&\nonumber \\&POS\{A\mathbf {X}\ge \widetilde{D}\}\ge \alpha , \end{aligned}$$

27 $$\begin{aligned}&POS\{B\mathbf {X}\le \widetilde{S}\mathbf {Y}\}\ge \beta , \end{aligned}$$

$$\begin{aligned} (15). \end{aligned}$$

Following a similar defuzzification approach, it can be re-written as,

28 $$\begin{aligned} \text {(OM2)\quad }\min \quad&\omega _1=F\mathbf {Y}+\left( \frac{C^1+C^2+C^3+C^4}{4}\right) \mathbf {X} \end{aligned}$$

29 $$\begin{aligned} \min \quad&\omega _2=G\mathbf {X} \end{aligned}$$

30 $$\begin{aligned} \text {s.t.}&\nonumber \\&A\mathbf {X}\ge (1-\alpha )D^1+\alpha D^2, \end{aligned}$$

31 $$\begin{aligned}&B\mathbf {X}\le \left( (1-\beta )S^4+\beta S^3\right) \mathbf {Y}, \end{aligned}$$

$$\begin{aligned} (15). \end{aligned}$$

Then, our novel combined possibility-necessity fuzzy model for controlling uncertain parameters and the fuzzy possibility-necessity PCCP model is obtained as formulated below,

32 $$\begin{aligned} \text {(OPM1)\quad }\min \quad&E[\omega _1]=F\mathbf {Y}+E[\widetilde{C}]\mathbf {X} \end{aligned}$$

33 $$\begin{aligned} \min \quad&\omega _2=G\mathbf {X} \end{aligned}$$

34 $$\begin{aligned} \text {s.t.}&\nonumber \\&(1-\nu _1)[NEC\{A\mathbf {X}\ge \widetilde{D}\}\ge \alpha ]+\nu _1[POS\{A\mathbf {X}\ge \widetilde{D}\}\ge \alpha ], \end{aligned}$$

35 $$\begin{aligned}&(1-\nu _2)[NEC\{B\mathbf {X}\le \widetilde{S}\mathbf {Y}\}\ge \beta ]+\nu _2[POS\{B\mathbf {X}\le \widetilde{S}\mathbf {Y}\}\ge \beta ], \end{aligned}$$

$$\begin{aligned} (15). \end{aligned}$$

In the above fuzzy possibility-necessity model $\nu _1$ and $\nu _2$ are binary parameters. If $\nu _1=\nu _2=1$ the model becomes an optimistic model while $\nu _1=\nu _2=0$ converts it to a pessimistic one. Therefore, by defining $\nu _1$ and $\nu _2$ as binary variables for controlling the fuzzy uncertain parameters, the following model is obtained,

36 $$\begin{aligned} \text {(OPM2)\quad }\min \quad&\omega _1=F\mathbf {Y}+\left( \frac{C^1+C^2+C^3+C^4}{4}\right) \mathbf {X} \end{aligned}$$

37 $$\begin{aligned} \min \quad&\omega _2=G\mathbf {X} \end{aligned}$$

38 $$\begin{aligned} \text {s.t.}&\nonumber \\&A\mathbf {X}\ge \Big ( (1-\nu _1) [(1-\alpha )D^3+\alpha D^4] +\nu _1[(1-\alpha )D^1+\alpha D^2] \Big ), \end{aligned}$$

39 $$\begin{aligned}&B\mathbf {X}\le \Big ((1-\nu _2) [(1-\beta )S^2+\beta S^1] +\nu _2[(1-\beta )S^4+\beta S^3]\Big )\mathbf {Y}, \end{aligned}$$

40 $$\begin{aligned}&(15),\nonumber \\&\nu _1,\nu _2\in \{0,1\}. \end{aligned}$$

In PCCP models, the minimum confidence level is determined according to the decision-maker’s preference and in the provided models the objective function is insensitive to this parameter. Nevertheless, obtaining robust solutions is not guaranteed and decision-makers may be exposed to high risks in their strategic decisions when robustness is in high demand. Thus, to avoid such circumstances, further a robust possibilistic-necessity counterpart model of our problem is proposed. This method was first introduced by Pishvaee et al. (2012) and it benefits both robust and possibilistic programming. We describe this method by applying it to our model below in (R-OPM).

41 $$\begin{aligned} \text {(R-OPM) }\min \text { } \omega _1^*=&E[\omega _1]\nonumber \\&+\zeta \big (f^1_{(max)}-E[\omega _1]\big )\nonumber \\&+M\Big [(1-\nu _1)(D^4-D^3)+\nu _1(D^2-D^1) +(1-\nu _2)(S^2-S^1)+\nu _2(S^4-S^3)\Big ]\nonumber \\&+\eta _1\Big [(1-\nu _1)(1-\alpha )(D^4-D^3)+ \nu _1\alpha (D^2-D^1)\Big ]\nonumber \\&+\eta _2\Big [(1-\nu _2)(1-\beta )(S^2-S^1)+ \nu _2\beta (S^4-s^3)\Big ]\mathbf {Y} \end{aligned}$$

42 $$\begin{aligned} \min \quad&\omega _2=G\mathbf {X} \end{aligned}$$

43 $$\begin{aligned} \text {s.t.}&\nonumber \\&A\mathbf {X}\ge \Big ( (1-\nu _1) [(1-\alpha )D^3+\alpha D^4]+ \nu _1[(1-\alpha )D^1+\alpha D^2] \Big ), \end{aligned}$$

44 $$\begin{aligned}&B\mathbf {X}\le \Big ((1-\nu _2) [(1-\beta )S^2+\beta S^1]+\nu _2[(1-\beta )S^4+\beta S^3]\Big )\mathbf {Y}, \end{aligned}$$

$$\begin{aligned} (15), (40),\quad \alpha ,\beta \in [0,1], \end{aligned}$$

where $f^1_{(max)}=F\mathbf {Y}+ C^4\mathbf {X}$ and *M* is a sufficiently large number. In the first objective function (41), the first term corresponds to the expected value of the uncertain parameters while the second one corresponds to the penalty for deviating from the desired value of the first objective (robustness of optimality) and the rest of them penalizes the unmet (uncertain) demand and also its excess from the capacity. Therefore, $\zeta$ is the coefficient of objective, $\eta _1$ denotes the unit cost of unsatisfied demand and $\eta _2$ is the unit penalty for excess use of the facilities. Parameters $\alpha$ and $\beta$ are the correction coefficients in fuzzy levels within the interval of [0, 1], which are decision variables in the model. Hence, the above given R-OPM model is nonlinear due to the terms $\alpha \nu _1$, $\beta \mathbf {Y}$, $\nu _2\mathbf {Y}$ and $\beta \nu _2 \mathbf {Y}$ in (43) and (44).

The multiplication of a binary and continuous variables like $\alpha \nu _1$ and can be linearized by replacing it with a non-negative continuous variable like $R^0$ together with three additional constraints to guarantee its appropriate value. The value of *M* in such a linearization is generally set as the upper bound of the continuous variable, which equals 1 here. Thus,

45a $$\begin{aligned}&(43) \Leftrightarrow&A\mathbf {X}\ge (1-\nu _1-\alpha +\alpha \nu _1)D^3+(\alpha -\alpha \nu _1) D^4+(\nu _1-\alpha \nu _1)D^1+\alpha \nu _1 D^2 \end{aligned}$$

45b $$\begin{aligned}\Leftrightarrow & {} A\mathbf {X}\ge D^3(1-\nu _1) +(D^4-D^3)\alpha +D^1\nu _1-(D^1-D^2-D^3+D^4)\alpha \nu _1 \end{aligned}$$

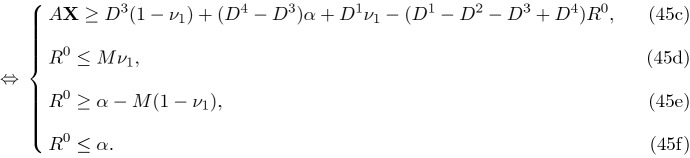


Also, to linearize a multiplication of two binary variables like $\nu _2\mathbf {Y}$ it suffices to replace it with an auxiliary binary variable, say *Z*, and add the following additional constraints (46–49).

46 $$\begin{aligned} \mathbf {Y}+\nu _2-Z\le 1, \end{aligned}$$

47 $$\begin{aligned} Z\le \mathbf {Y}, \end{aligned}$$

48 $$\begin{aligned} Z\le \nu _2, \end{aligned}$$

49 $$\begin{aligned} Z\in \{0,1\}. \end{aligned}$$

Therefore, (44) can also be reformulated in a linear form using *Z*,$R^1$ and $R^2$ instead of the nonlinear terms as shown below.

50a $$\begin{aligned} (44)\Leftrightarrow&B\mathbf {X}\le [(1-\beta )S^2+\beta S^1]\mathbf {Y}-[(1-\beta )S^2+\beta S^1]\nu _2\mathbf {Y}+[(1-\beta )S^4+\beta S^3]\nu _2\mathbf {Y} \end{aligned}$$

50b $$\begin{aligned} \Leftrightarrow&B\mathbf {X}\le [(1-\beta )S^2+\beta S^1]\mathbf {Y}-[(1-\beta )S^2+\beta S^1]Z+[(1-\beta )S^4+\beta S^3]Z \end{aligned}$$

50c $$\begin{aligned} \Leftrightarrow&B\mathbf {X}\le [S^2+\beta ( S^1-S^2)]\mathbf {Y}-[S^2+\beta (S^1-S^2)]Z+[(S^4+\beta (S^3-S^4)]Z \end{aligned}$$

50d $$\begin{aligned} \Leftrightarrow&B\mathbf {X}\le S^2\mathbf {Y}+ (S^1-S^2)\beta \mathbf {Y} + (S^4-S^2)Z-( S^1-S^2-S^3+S^4)\beta Z. \end{aligned}$$

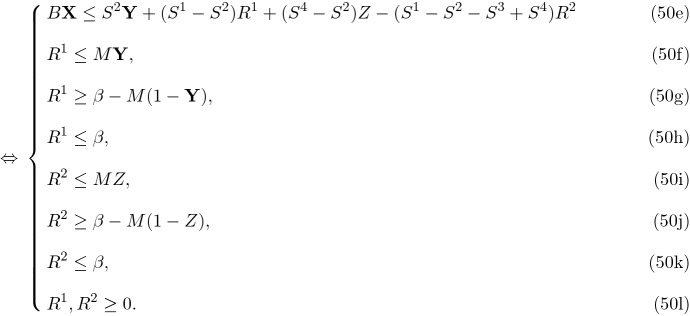


### The linear programming model of blood supply chain network

Based on the above discussed methodology and the basic BSC model, the linearized version of the robust fuzzy possibilistic model for the blood supply chain network design is presented in (51–80).

51 $$\begin{aligned} \text {(LRFP) }\min \omega ^*_1=&E[\omega _1]+\zeta \big (f^1_{(max)}-E[\omega _1]\big ) +M\sum \limits _{g\in \mathcal {G}}\Bigg [\nonumber \\&\sum \limits _{h\in \mathcal {H}}\sum \limits _{t\in \mathcal {T}} \Big [(1-\nu _1)(d^4_{hgt}-d^3_{hgt})+\nu _1(d^2_{hgt}-d^1_{hgt})\Big ]\nonumber \\&+\sum \limits _{j\in \mathcal {J}} \Big [(1-\nu _2)(c^{C,2}_{jg}-c^{C,1}_{jg})+\nu _2(c^{C,4}_{jg}-c^{C,3}_{jg})\Big ]\nonumber \\&+\sum \limits _{k\in \mathcal {K}} \Big [(1-\nu _3)(c^{L,2}_{kg}-c^{L,1}_{kg})+\nu _3(c^{L,4}_{kg}-c^{L,3}_{kg})\Big ]\Bigg ]\nonumber \\&+\sum \limits _{g\in \mathcal {G}}\Bigg [ \eta _1\sum \limits _{h\in \mathcal {H}}\sum \limits _{t\in \mathcal {T}} \Big [\underbrace{(1-\nu _1-\alpha +R^0}_{(1-\nu _1)(1-\alpha )})(d^4_{hgt}-d^3_{hgt})+ R^0(d^2_{hgt}-d^1_{hgt})\Big ]\nonumber \\&+\eta _2\sum \limits _{j\in \mathcal {J}} \Big [(\underbrace{Y^C_j-R^{C,1}_j-Z^1_j+R^{C,2}_j}_{(1-\nu _2)(1-\beta )Y^C_j})(c^{C,2}_{jg}-c^{C,1}_{jg})+ R^1_j(c^{C,4}_{jg}-c^{C,3}_{jg})\Big ]\nonumber \\&+\eta _3\sum \limits _{k\in \mathcal {K}} \Big [(\underbrace{Y^L_k-R^{L,1}_k-Z^2_k+R^{L,2}_k}_{(1-\nu _3)(1-\beta )Y^L_k})(c^{L,2}_{kg}-c^{L,1}_{kg})+R^2_k(c^{L,4}_{kg}-c^{L,3}_{kg})\Big ] \Bigg ] \end{aligned}$$

52 $$\begin{aligned} \min \omega _2=&\sum \limits _{t\in \mathcal {T}}\sum \limits _{g\in \mathcal {G}}\Big [ \sum _{i,j}e^1_{ij}X_{ijgt} +\sum \limits _{r=r_0}^t\Big (\sum _{j,k}e^2_{jk}U_{jkgtr}+ \sum _{k',k}e^3_{k'k}V_{k'kgtr}+\sum _{k,h}e^4_{k'k}S_{khgtr}\Big )\Big ] \end{aligned}$$

53 $$\begin{aligned}&\text {s.t.}\nonumber \\ E[\omega _1]&=\sum \limits _{j\in \mathcal {J}}^{}f^C_j Y^C_j+\sum \limits _{k\in \mathcal {K}}^{}f^L_k Y^L_k +\sum \limits _{t\in \mathcal {T}}\sum \limits _{g\in \mathcal {G}}\Bigg \{ \quad \sum _{i,j}\left( \frac{\theta ^{1,1}_{ij}+\theta ^{1,2}_{ij}+\theta ^{1,3}_{ij}+\theta ^{1,4}_{ij}}{4}\right) X_{ijgt}\nonumber \\&+\sum \limits _{r=r_0}^t\Bigg [ \sum _{j,k}\left( \frac{\theta ^{2,1}_{jk}+\theta ^{2,2}_{jk}+\theta ^{2,3}_{jk}+\theta ^{2,4}_{jk}}{4}\right) U_{jkgtr} +\sum _{k',k}\left( \frac{\theta ^{3,1}_{k'k}+\theta ^{3,2}_{k'k}+\theta ^{3,3}_{k'k}+\theta ^{3,4}_{k'k}}{4}\right) V_{k'kgtr}\nonumber \\&+\sum _{k,h}\left( \frac{\theta ^{4,1}_{kh}+\theta ^{4,2}_{kh}+\theta ^{4,3}_{kh}+\theta ^{4,4}_{kh}}{4}\right) S_{khgtr}\Bigg ] +\sum \limits _{r=r_0}^t\Big (\sum \limits _{j\in \mathcal {J}}h^C_j I^C_{jgtr}+\sum \limits _{k\in \mathcal {K}}h^L_k I^L_{kgtr}\Big )\nonumber \\&+\sum \limits _{h\in \mathcal {H}}\pi _{hgt}B_{hgt} \Bigg \}, \end{aligned}$$

54 $$\begin{aligned} f^1_{(max)}&=\sum \limits _{j\in \mathcal {J}}^{}f^C_j Y^C_j+\sum \limits _{k\in \mathcal {K}}^{}f^L_k Y^L_k +\sum \limits _{t\in \mathcal {T}}\sum \limits _{g\in \mathcal {G}}\Bigg \{ \sum _{i,j}\theta ^{1,4}_{ij}X_{ijgt}\nonumber \\&+\sum \limits _{r=r_0}^t\Big [\sum _{j,k}\theta ^{2,4}_{jk}U_{jkgtr}+\sum _{k',k}\theta ^{3,4}_{k'k}V_{k'kgtr}+\sum _{k,h}\theta ^{4,4}_{kh}S_{khgtr}\Big ]\nonumber \\&+\sum \limits _{r=r_0}^t\Big (\sum \limits _{j\in \mathcal {J}}h^C_j I^C_{jgtr}+\sum \limits _{k\in \mathcal {K}}h^L_k I^L_{kgtr}\Big )+\sum \limits _{h\in \mathcal {H}}\pi _{hgt}B_{hgt} \Bigg \}, \end{aligned}$$

55 $$\begin{aligned}&(3),(4),\nonumber \\&\sum \limits _{i\in \mathcal {I}} X_{ijgr}\le c^{C,2}_{jg}Y^C_j+ (c^{C,1}_{jg}-c^{C,2}_{jg})R^{C,1}_j+\nonumber \\&\quad \qquad \qquad (c^{C,4}_{jg}-c^{C,2}_{jg})Z^1_j-(c^{C,1}_{jg}-c^{C,2}_{jg}-c^{C,3}_{jg}+c^{C,4}_{jg}) R^{C,2}_j,\quad \forall j,g,r, \end{aligned}$$

56 $$\begin{aligned}&\sum \limits _{j\in \mathcal {J}}\sum \limits _{r=r_0}^t U_{jkrt}+ \sum \limits _{k'\in \mathcal {K}}\sum \limits _{r=r_0}^t V_{k'kgtr}\le c^{L,2}_{kg} Y^L_k+ (c^{L,1}_{kg}-c^{L,2}_{kg})R^{L,1}_k+\nonumber \\&\qquad \qquad \quad (c^{L,4}_{kg}-c^{L,2}_{kg})Z^2_k -(c^{L,1}_{kg}-c^{L,2}_{kg}-c^{L,3}_{kg}+c^{L,4}_{kg})R^{L,2}_k, \quad \forall k,g,t, \end{aligned}$$

57 $$\begin{aligned}&\sum \limits _{k\in \mathcal {K}}\sum \limits _{r=r_0}^t S_{khgtr}+B_{hgt}\ge d^3_{hgt}(1-\nu _1) +(d^3_{hgt}-d^4_{hgt})\alpha +d^1_{hgt}\nu _1\nonumber \\&\qquad \qquad \quad \quad \qquad \qquad -(d^1_{hgt}-d^2_{hgt}-d^3_{hgt}+d^4_{hgt})R^0,\qquad \forall h,g,t, \end{aligned}$$

58 $$\begin{aligned}&R^0\le M\nu _1, \end{aligned}$$

59 $$\begin{aligned}&R^0\ge \alpha -M(1-\nu _1), \end{aligned}$$

60 $$\begin{aligned}&R^0\le \alpha , \end{aligned}$$

61 $$\begin{aligned}&Y^C_j+\nu _2-Z^1_j\le 1,\qquad \forall j, \end{aligned}$$

62 $$\begin{aligned}&Z^1_j\le Y^C_j,\qquad \forall j, \end{aligned}$$

63 $$\begin{aligned}&Z^1_j\le \nu _2,\qquad \forall j, \end{aligned}$$

64 $$\begin{aligned}&R^{C,1}_j\le MY^C_j,\qquad \forall j, \end{aligned}$$

65 $$\begin{aligned}&R^{C,1}_j\ge \beta -M(1-Y^C_j),\qquad \forall j, \end{aligned}$$

66 $$\begin{aligned}&R^{C,1}_j\le \beta , \qquad \forall j, \end{aligned}$$

67 $$\begin{aligned}&R^{C,2}_j\le M Z^1_j,\qquad \forall j, \end{aligned}$$

68 $$\begin{aligned}&R^{C,2}_j\ge \beta -M(1-Z^1_j),\qquad \forall j, \end{aligned}$$

69 $$\begin{aligned}&R^{C,2}_j\le \beta ,\qquad \forall j, \end{aligned}$$

70 $$\begin{aligned}&Y_k+\nu _3-Z^2_k\le 1,\qquad \forall k, \end{aligned}$$

71 $$\begin{aligned}&Z^2_k\le Y^L_k,\qquad \forall k, \end{aligned}$$

72 $$\begin{aligned}&Z^2_k\le \nu _3,\qquad \forall k, \end{aligned}$$

73 $$\begin{aligned}&R^{L,1}_k\le M Y^L_k,\qquad \forall k, \end{aligned}$$

74 $$\begin{aligned}&R^{L,1}_k\ge \beta -M(1-Y^L_k),\qquad \forall k, \end{aligned}$$

75 $$\begin{aligned}&R^{L,1}_k\le \beta ,\qquad \forall k, \end{aligned}$$

76 $$\begin{aligned}&R^{L,2}_k\le MZ^2_k,\qquad \forall k, \end{aligned}$$

77 $$\begin{aligned}&R^{L,2}_k\ge \beta -M(1-Z^2_k),\qquad \forall k, \end{aligned}$$

78 $$\begin{aligned}&R^{L,2}_k\le \beta , \end{aligned}$$

79 $$\begin{aligned}&(9),(10),\text { }\alpha ,\beta \in [0,1],\nonumber \\&\nu _1,\nu _2,\nu _3,Z^1_j,Z^2_j\in \{0,1\},\qquad \forall j, \end{aligned}$$

80 $$\begin{aligned}&R^0,R^{C,1}_j,R^{C,2}_j,R^{L,1}_k,R^{L,2}_k\ge 0,\qquad \qquad \forall k\in \mathcal {K},j\in \mathcal {J}. \end{aligned}$$

The objective functions (51) and (52) have been linearized following the process depicted in the previous subsection. Thus, inequalities (58–78) correspond to those auxiliary constraints needed for linearization of the nonlinear terms similar to (45d)–(45f) and (50f)–(50l). Constraints (53) and (54) accommodate computation of the two terms defined in the objective function of R-OPM (41), namely, $f^1_{(max)}$ and $E[\omega _1]$. Constraint (55) restricts the daily amount of blood donation due to the capacities in for each blood type in donation centers and/or force to the model to open a donation center. Similarly, constraint (56) limits the daily number of received blood bags in each lab based on the capacity and let the model open a lab if needed. Constraint (57) deals with satisfying the demand which is constructed following (43).

## Multi-objective solution methods

Because the objective functions are in trade-off, a multi-objective decision making method should be employed. The LP-metric, Goal-Programming, and Torabi-Hassini methods are employed and briefly described in the following. The common feature of these three particularly chosen methods is that they all rest on minimization of some sort of deviations from individual desired goals and therefore it is worth seeing their difference in the results.

### LP-metric (LP) method

In this method, the objective functions of the mathematical programming model converted to a single objective, which minimizes the total distance of each individual objective from its ideal value. This is shown in the following,

81 $$\begin{aligned} \min D=\left[ \sum _{k}\gamma _k\left| \frac{w^*_k-w_k}{w^*_k}\right| ^p\right] ^{1/p} \end{aligned}$$

where $1\le p\le \infty$, $w_k$ and $w^*_k$ are respectively the* k*th objective function and its individual ideal objective value.

### Goal programming (GP) method

This method (see Hwang and Masud (2012)), minimizes a weighted aggregate deviation of the objectives functions from their goals as shown below,

82 $$\begin{aligned} \min D&=\sum _{k}\gamma _k h(\rho ^+,\rho ^-) \end{aligned}$$

83 $$\begin{aligned}&\text {s.t.}\nonumber \\&w_k-w^*_k=\rho ^+-\rho ^1, \end{aligned}$$

84 $$\begin{aligned}&\rho ^+,\rho ^-\ge 0, \end{aligned}$$

where $\rho ^+$ and $\rho ^-$ are excess and slack from the goal; $\gamma _k$ is the weight associated with the* k*th objective and the distance function *h* is defined as,

85 $$\begin{aligned} h(\rho ^+,\rho ^-)=\left\{ \begin{array}{ll} \rho ^+,&{}\text { if }w_k,\text { is maximization,} \\ \rho ^-,&{}\text { if }w_k,\text { is minimization,} \\ \rho ^++\rho ^-,&{}\text { otherwise. } \\ \end{array} \right. \end{aligned}$$

### Torabi-Hassini (TH) method

This method (see Torabi and Hassini 2008) also rests on minimization of the weighted total deviation of objectives from their goals. However, in lieu of the absolute deviation, a normalized membership function given in (86), is used. In this function, $w_k^{PIS}$ and $w_k^{NIS}$ are the positive and negative ideal solutions of each objective which are obtained by a single objective minimization or maximization model for each objective, separately.

86 $$\begin{aligned} \mu _k(x)\left\{ \begin{array}{ll} 1&{}\text { if } w_k<w_k^{PIS},\\ \frac{w_k^{NIS}-w_k}{w_k^{NIS}-w_k^{PIS}}&{}\text { if } w_k^{PIS}\le w_k\le w_k^{NIS},\\ 0&{} \text { if } w_k>w_k^{NIS}, \end{array} \right. \end{aligned}$$

where $\mu (x)$ indicates the satisfaction level of the $k\text {th}$ objective function for a given solution *x*. Let $\vartheta _0$ be the minimum satisfaction level of objectives, $\vartheta _0=\min _k\{\mu _k(x)\}$ and *F* denote the feasible region of the original model. Then the following problem is solved to integrate the objectives as,

87 $$\begin{aligned} \max \text { } \vartheta (x)&=\phi \vartheta _0+(1-\phi )\sum _{k}\gamma _k\mu _k(x) \end{aligned}$$

88 $$\begin{aligned}&\text {s.t.}\nonumber \\&\vartheta _0\le \mu _k(x),\qquad \forall k, \end{aligned}$$

89 $$\begin{aligned}&x\in F,\quad \vartheta _0 \text { and }\phi \in [0,1]. \end{aligned}$$

The parameter $\phi$ controls the minimum satisfaction level of objectives and the compromise degree among the objectives as well.

## Numerical analysis

**Fig. 2:**
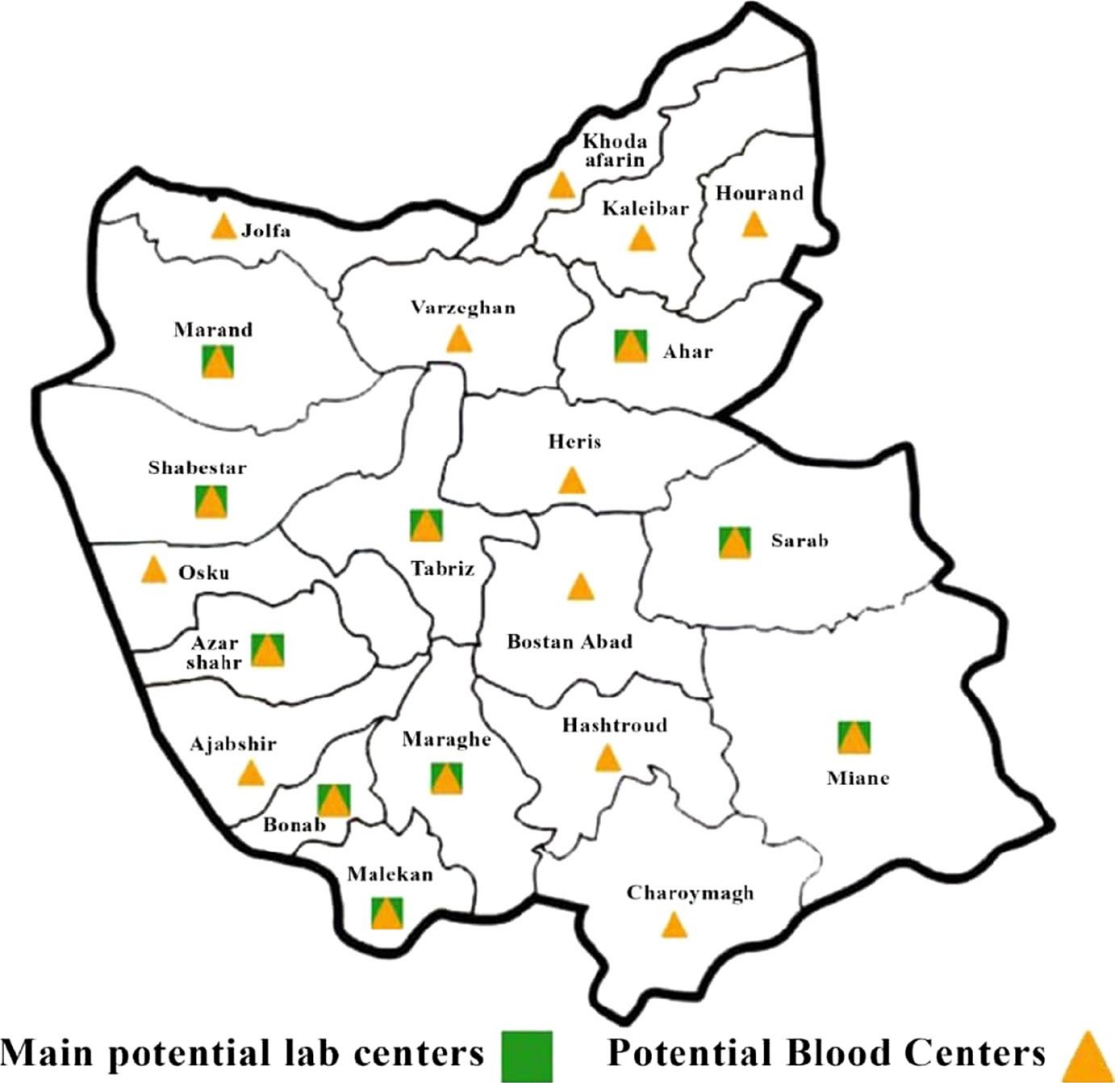
Potential donation centers and labs

A real case of blood supply chain network design has been used to validate this model, employing the three aforementioned methods of multi-objective decision making in order to compare them. Our network includes 21 cities in a northern province in Iran, all 8 blood groups (A$^+$,A$^-$,B$^+$,B$^-$,AB$^+$,AB$^-$,O$^+$,O$^-$) in a 12 months planning horizon. Furthermore, all of the 21 cities of this province were considered as potential donation and demand points while only 10 biggest cities among them can accommodate the laboratory locations as shown in Fig. 2.

### Parameter setting

The distance matrix of the potential facility location was obtained by pairwise querying between their coordination on Google maps. The parameters regarding blood demand were set according to the local authority (https://tabriz.ibto.ir). The upper and lower bounds of the parameters have been estimated by expert opinion and the uncertain intervals were set accordingly. In addition, a range of weights in the interval [0.1, 0.9] are assigned for the objective functions and the corresponding results are tabulated in the next subsection. However, only the equally weighted objectives are illustrated in the figures. The numerical values corresponding to our parameters are summarized in Table 4 and the pairwise distance of locations is provided in Table 11 of the Appendix. All the time units are represented in seconds (s) and all cost-related numbers are in $0.04 while the emission-related numbers, unless specified, are in 0.1KgCO$$_2$$e.

**Table 4:** Parameter setting for the numerical study

Parameter	Values			
Deterministic parameters:				
$f^C_j$	$\sim U(60000,80000)$			
$f^L_k$	$\sim U(120000,150000)$			
$h^C_j,h^L_j$	$\sim U(2,3)$			
$v_g$	$\sim U(1,5)$			
$\pi _{hgt}$	$\sim U(100,200)$			
$e^1_{ij},e^2_{jk},e^3_{k'k},e^4_{kh}$	$0.80\delta _{..}$			
Fuzzy trapezoidal parameters: $\tilde{a}=(a_1,a_2,a_3,a_4)$				
$\tilde{a}$	$a_1$	$a_2$	$a_3$	$a_4$
$\widetilde{c}^C_{jg}$:	$\sim U(900,1000)$	$\sim U(1000,1100)$	$\sim U(1100,1200)$	$\sim U(1200,1300)$
$\widetilde{c}^L_{kg}$:	$\sim U(1400,1500)$	$\sim U(1500,1600)$	$\sim U(1600,1700)$	$\sim U(1700,1800)$
$\widetilde{d}_{hgt}$:	$\sim U(80,100)$	$\sim U(100,120)$	$\sim U(120,140)$	$\sim U(140,160)$
$\widetilde{\theta }^1_{ij}$, $\widetilde{\theta }^2_{jk}$, $\widetilde{\theta }^3_{k'k}$, $\widetilde{\theta }^4_{kh}$:	$0.90\omega \delta _{..}$	$0.95\omega \delta _{..}$	$1.05\omega \delta _{..}$	$1.10\omega \delta _{..}$

$\delta _{..}$: distance of locations with the associated indices; $\omega =0.888$ : per distance unit cost

### Computational results

Using GAMS Software version 24.8.5 on a desktop computer equipped with Intel(R) Core(TM) i7-4710HQ and 8GB of RAM, the best and worst values of each objective and their corresponding computation time are obtained and listed in Table 5.

**Table 5:** Objective values for individual objective functions

	Best objective	Worst objective	Computational time (s)
Objective 1	16596489.76	37767006.41	563
Objective 2	2797559.31	3899116.19	45

**Fig. 3:**
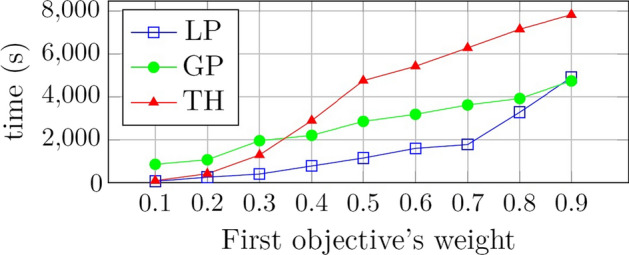
Comparison of computation times among the methods

Figure 3 shows the sensitivity of the computational times to the weight of the first objective function for each solution method. As shown, the TH method is computationally more expensive and sensitive to the weight. When the weights are increased, the computation time of the GP method increases with a smooth slope compared to other solution methods.

**Fig. 4:**
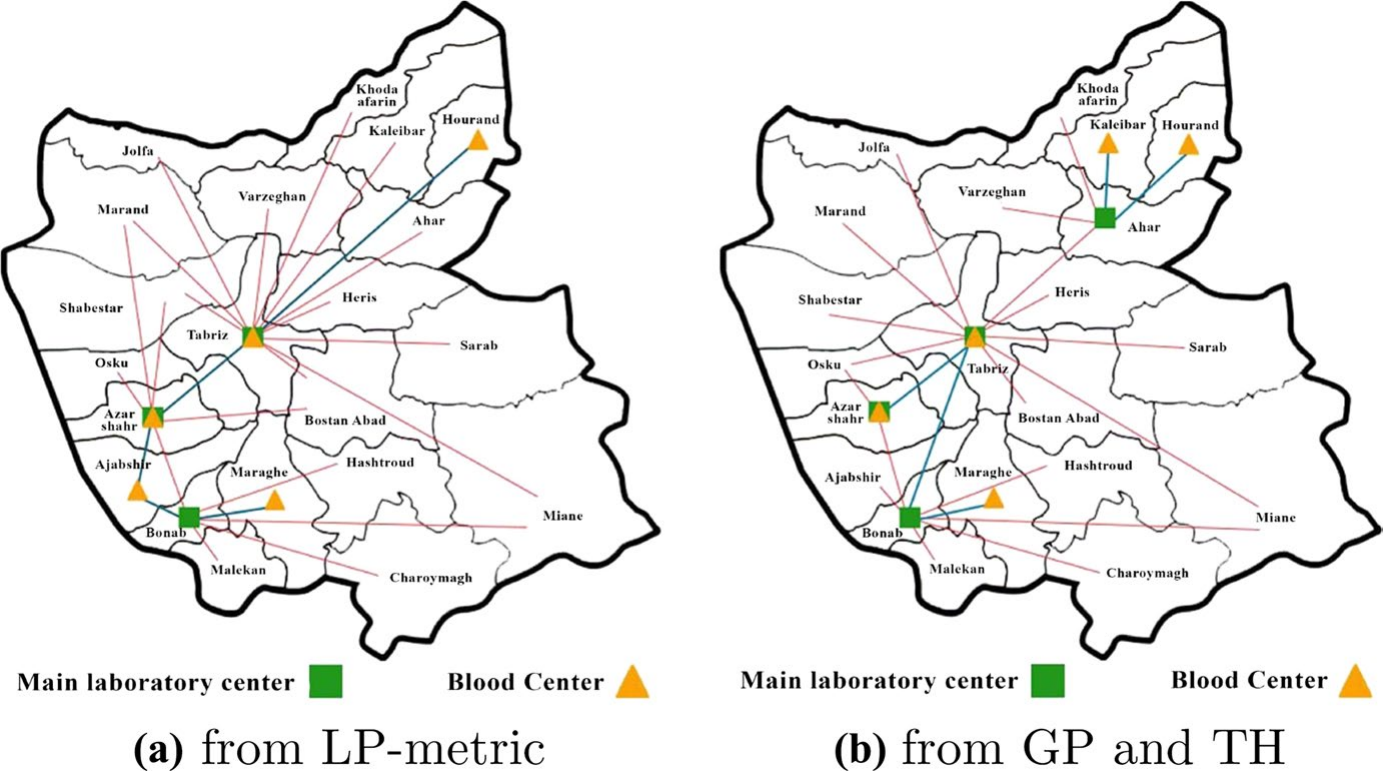
The chosen donation centers and labs for equally weighted objectives

Table 6 shows the result of the LP-metric ($p=1$), GP, and TH multi-objective methods, respectively. The values of each objective function as well as the computation times are summarized for different objective weights from the decision-makers viewpoint. According to this table, when the weight of the first objective function is increased its value decreases and its computational time increases. The average value of the first objective is better in GP method compared to other approaches, whereas for the second objective function, the TH method has resulted in a better averaged value. The chosen locations for establishment of blood centers and labs, obtained from the solution of equally weighted (0.5) objectives in each method, are illustrated in Fig. 4. The red lines in these figures correspond to distribution of blood bags from laboratories to demand points while the blue lines show the allocation of the labs to the donation centers. Although the objective values are different, the GP and TH methods have resulted in the same network for the objective weight values of 0.5 as shown in Fig. 4b.

**Table 6:** The results of three multi-objective methods

Weight of obj. 1	LP-metric			GP			TH		
	Objective value 1	Objective value 2	Comp. time	Objective value 1	Objective value 2	Comp. time	Objective value 1	Objective value 2	Comp. time
0.1	30752901.3	2798611.5	67	19472695.7	3245890.2	859	30762063.3	2798611.5	96
0.2	21079933.2	3142251.8	258	17999123.3	3493150.2	1071	30756732.5	2798618.4	419
0.3	19604562.0	3230909.2	405	16744305.8	3742965.9	1959	30752907.9	2798621.0	1295
0.4	18148064.4	3380668.4	781	16743073.3	3743741.4	2209	21816642.8	3119676.3	2898
0.5	18017065.1	3395416.0	1152	16742904.1	3743862.9	2860	21079396.1	3143036.6	4759
0.6	18016981.8	3395986.9	1602	16603984.2	3860288.8	3189	18545250.4	3439829.8	5429
0.7	16744304.7	3742965.9	1778	16602486.3	3862566.3	3625	17642867.0	3628298.2	6286
0.8	16743072.6	3743741.4	3287	16601514.0	3865959.9	3927	17365428.3	3696248.4	7156
0.9	16603982.6	3860288.8	4911	16597311.1	3888264.4	4745	16601694.7	3865478.6	7836
Average	19523429.7	3410093.3	1582	17123044.2	3716298.9	2716	22813664.8	3254268.7	4019

**Fig. 5:**
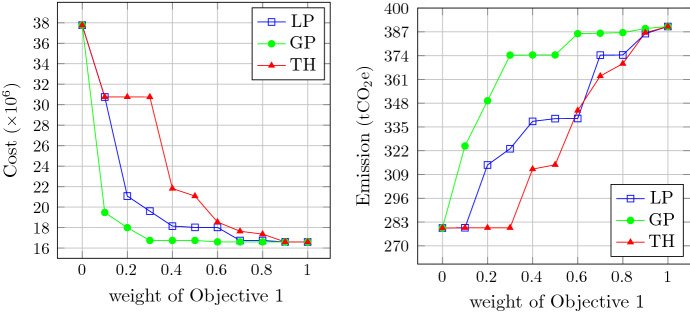
Comparison of objective values of LP-metric, GP and TH methods over different weights

**Fig. 6:**
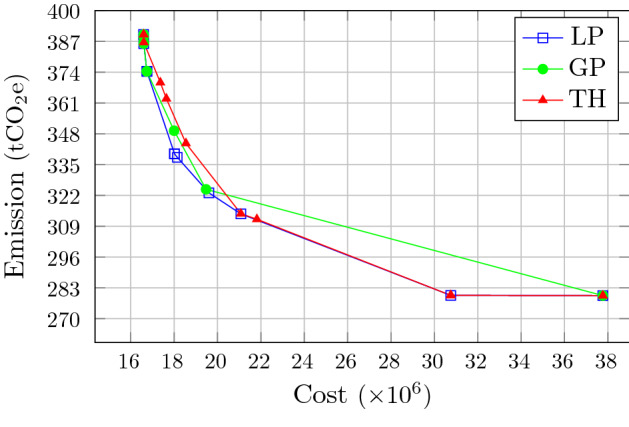
Comparison of Pareto frontiers among the methods for equally weighted objectives

The trade-off between the objective functions is detectable in all methods. According to Table 6, consideration of the second objective from the minimum to maximum assigned weight has caused the carbon emission to reduce by more than 27.5%, 16.5 and 27.6% in LP-metric, GP and TH methods, respectively. By the extreme values of objective weights (i.e., 0 and 1), these methods converge to the values given in Table 5. As seen there, solely optimizing the model with respect to the second objective leads to 28.3% less carbon emission compared to the case of purely cost optimization. The trends of optimum values obtained by each method for both objective functions are illustrated in Figure 5, wherein the first and last points are overlapping, because when the weights equal 0 or 1 in fact one objective is left to be optimized, and all these methods lead to the same solution. This trade-off is illustrated for equally weighted objectives for each method using the Pareto-frontier form in Figure 6. The horizontal axis in this figure corresponds to the first objective (operational cost) and the vertical axis represents the second one (carbon footprint). As shown in this graph, the spread of objective values is not varied for different methods, and the range of the objectives obtained from GP method takes place in a more compact interval compared to the other methods. Therefore, it indicates that LP-metric and TH solution methods provide the decision-makers with a more diverse set of solutions.

Besides, six measures are used to assess these methods in efficiency of their solution: (i) The maximum spread index (MSI) (Zitzler et al. 2000), (ii) The spacing metric (SM) (Schott 1995), (iii) The number of Pareto front (NPF) (Deb and Jain 2002), (iv) The mean ideal distance (MID) which quantifies the convergence of a solution approach (see Karimi et al. 2010), (v) The mean of objective functions (MOBF) for both objectives (see Szmelter-Jarosz et al. 2021) and finally (vi) The CPU-Time. The method with bigger values of MSI and NPF, and smaller values of SM, MID, CPU-Time, MOBF measures is proven to be more efficient. These measures are tabulated in Table 7.

**Table 7:** Comparison of multi objective methods with respect to six measures

Method	MSI	SM	MID	NPF	MOBF1	MOBF2	CPU-TIME(s)
LP	14188694.7	1.329	0.710	9	19523429.7	3410093.3	1582.3
GP	2946265.6	1.605	0.870	9	17123044.2	3716298.9	2716.0
TH	14200501.6	0.756	0.796	9	22813664.8	3254268.7	4019.3

Although the above discussion shows the difference between the objective values for each solution method, the question remains on how statistically valid and meaningful they are. Thus, five replications randomly drawn from the distributions given in Table 4 were set to conduct the Tukey multiple comparison test in order to address this concern, and the results for 95% confidence level is summarized in Table 8. This Table shows that the differences between objective values are statistically meaningful (p-value less than 0.05) and therefore, verifies the above result which concludes TH method offers the most efficient solution, while the LP-metric solution provides the least computationally expensive solution and the best Pareto frontier.

**Table 8:** Tukey’s multiple comparison results for 95% confidence level

Compared methods	Objective 1			Objective 2		
	Average difference	95% C-I	*P*-Value	Average difference	95% C-I	P-Value
LP-GP	2400386	(−1108934, 5909705)	0.153	306206	(593267, 19144)	0.038
LP-TH	3290235	(−8743581, 2163111)	0.217	155825	(−225166, 536815)	0.397
GP-TH	5690621	(10508923, 872318)	0.026	462030	(117522,806539)	0.013

Thus, in order to prioritize and select the most efficient solution method with respect to the above-mentioned performance indices, a ranking is also provided by the TOPSIS approach. The weights of the indices was assigned by Shannon’s entropy method. Accordingly, the corresponding utility and rank of each method determined as given in Table 9.

**Table 9:** Ranking of the multi-objective methods by TOPSIS

Method	Utility weight	Rank
LP	0.8917	1
GP	0.1568	3
TH	0.7398	2

### Sensitivity analysis

In order to investigate the changes in the uncertainty rate on the values of the objective functions (cost and emission), a sensitivity analysis has been performed. To that end, the uncertainty rates ($\alpha$ and $\beta$) were considered as a parameter where their values are between 0.1 and 0.9. As a result, the value of the obtained objective functions is shown in Table 10. From the managerial perspective, what we can read from these results is that with the increase of demand uncertainty rate, the costs related to network design have increased by 40.34% compared to the optimal state under certain situations. Also, due to the increase in the volume of blood products to be transferred to the demand points, the amount of carbon emissions has also increased.

**Fig. 7:**
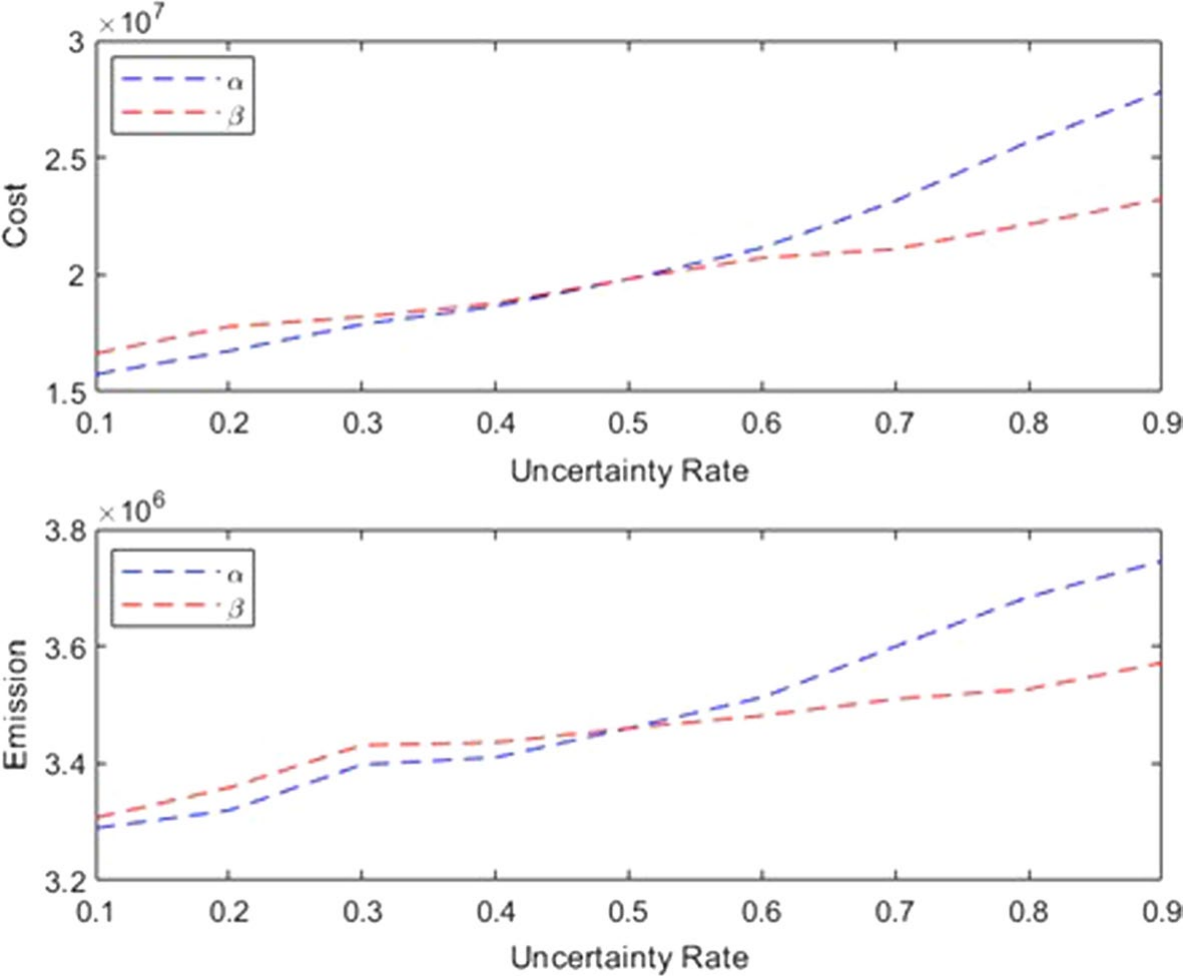
The trend of optimal objective values at different uncertainty levels ($\alpha$: demand uncertainty, $\beta$: capacity uncertainty

Additionally, as the uncertainty level in the capacity of potential facilities decreases it necessitates more centers to be built, and at greater distances from the demand points, and therefore the costs related to transportation and construction of centers will increase. Similarly the carbon footprint increases due to longer distances between facilities as well as more frequent blood product dispatches compared to the optimal state under less uncertainty. The total cost is increased by 17.14% compared to its optimal certain counterpart. Figure 7 shows the pictorial comparison of these trends.

**Table 10:** Objective values in the different uncertainty levels

$\beta =0.5$			$\alpha =0.5$		
$\alpha$	Obj.1	Obj. 2	$\beta$	Obj. 1	Obj. 2
0.1	15744315.6	3289512.2	0.1	16639541.7	3307143.2
0.2	16742154.2	3319542.8	0.2	17783347.8	3358441.7
0.3	17882103.7	3397854.1	0.3	18198732.3	3431148.5
0.4	18647946.2	3410168.5	0.4	18780425.2	3435747.4
0.5	19820046.2	3460220.3	0.5	19820046.2	3460220.3
0.6	21147658.5	3512683.6	0.6	20715657.6	3481548.2
0.7	23147551.6	3599746.4	0.7	21094508.6	3509826.0
0.8	25674988.5	3684520.0	0.8	22165115.2	3526798.7
0.9	27816541.3	3745412.3	0.9	23217275.1	3571898.6

## Conclusions

Designing an efficient blood supply chain at operational level corresponding to the high-level strategic objectives is a challenging task, because the product is highly perishable. It is therefore often dispatched to local regions with limited distances in order to keep certain transit lead-times within the shelf-time of the product. Such supply chain design and allocation problems can be extremely complicated, depending on the population distribution, as well as the number of demand points, donation points, and blood testing laboratories in the target regions.

The addressed BSC problem in this paper was modelled by a bi-objective optimization model to minimize both cost as well as carbon footprint under demand, donation and operational cost uncertainty. To incorporate uncertain parameters in the mathematical model a novel robust possibilistic-necessity approach was employed. The uncertain parameters first formulate in a fuzzy framework and then the concept was combined with a robust optimization technique. To manage multiple objectives three well-known approaches, namely, LP metric, Goal-Programming and Torabi-Hassini methods were examined, compared and ranked with respect to several measures such as MSI, SM, MID, NPF, MOBF1, MOBF2 and CPU-Time. The Pareto frontier as well as TOPSIS multi-criteria analysis on the mentioned measures determined LP method as the first ranked approach. According to our analysis, the LP and TH methods deliver more diverse non-dominant solutions than GP does, while for equally weighted objectives GP and TH lead to a similar supply chain design despite their different objective values.

Our numerical study showed that the worst case demand scenario imposes almost 40% more cost compared to its best counterpart, whilst the similar analysis over the capacity scenarios demonstrated a 17% difference. Thus, from the managerial perspective, it provides a figure on how much more investment is needed to prepare the infrastructure for the extreme cases or somewhere in between of the spectrum based on the optimism of the decision makers and the available budget.

Our work, however, has some limitations that can be addressed in future studies. Among those, compatibility of different donor and receiver blood groups can explicitly be considered and analysed rather than a net separate demand for each. That would add a additional dimension of complexity to the model but might capture more operational challenges of the real case. Furthermore, as the blood products are perishable, excess of the supply may lead to waste which is not only an important measure but also may have environmental drawbacks in line with the second objective of our model. Additionally, in terms of dealing with multiple objective, only three of the existing methods were examined, while other approaches such as Goal attainment, epsilon-constraint, maxi-min or weighted sum can also be investigated and compared in future studies.

## Appendix

See Table 11.

**Table 11:** Distances (in Km) matrix of the towns by which the supply network is defined in the case study

	Azarshahr	Mianeh	Sarab	Marand	Charoymaq	Bostan Abad	Shabestar	Varzeqan	Heris	Maragheh	Tabriz
Azarshahr	0										
Mianeh	222	0									
Sarab	187	98	0								
Marand	123	239	205	0							
Charoymaq	218	87	184	235	0						
Bostan Abad	117	106	71	134	113	0					
Shabestar	120	236	202	64	232	131	0				
Varzeqan	147	62	60	161	214	117	62	0			
Heris	147	175	81	161	183	74	131	104	0		
Maragheh	82	176	226	200	107	156	197	223	224	0	
Tabriz	63	174	134	70	171	63	129	98	90	139	0
Ahar	190	236	133	183	256	102	214	46	61	249	120
Kaleybar	249	299	193	243	316	162	274	228	121	358	180
Hashtrood	177	84	132	194	61	62	191	62	131	99	129
Osku	40	195	162	105	191	92	93	123	122	118	36
Jolfa	186	301	268	67	298	197	126	145	228	263	132
Khoda Afarin	294	345	239	289	381	208	309	154	167	371	237
Hurand	61	164	189	239	84	158	236	104	118	305	176
Malekan	88	197	248	206	129	177	120	232	230	43	146
Bonab	63	194	245	180	126	174	117	208	205	21	121
Ajabshir	41	216	222	158	148	151	155	181	182	42	97

	Ahar	Kaleybar	Hashtrood	Osku	Jolfa	Khoda Afarin	Hurand	Malekan	Bonab	Ajabshir
Azarshahr										
Mianeh										
Sarab										
Marand										
Charoymaq										
Bostan Abad										
Shabestar										
Varzeqan										
Heris										
Maragheh										
Tabriz										
Ahar	0									
Kaleybar	63	0								
Hashtrood	200	223	0							
Osku	145	205	150	0						
Jolfa	193	252	258	159	0					
Khoda Afarin	109	46	320	269	298	0				
Hurand	58	44	220	201	302	89	0			
Malekan	254	314	120	124	268	377	310	0		
Bonab	230	290	117	98	244	352	286	24	0	
Ajabshir	223	283	138	76	220	329	279	48	23	0
